# Clinical experience with medical hypnosis as an adjunctive therapy in heart surgery

**DOI:** 10.3389/fpsyg.2024.1356392

**Published:** 2024-02-19

**Authors:** Katharina Tigges-Limmer, Yvonne Brocks, Yvonne Winkler, Scott Stock Gissendanner, Jan Gummert

**Affiliations:** ^1^Department of Thoracic and Cardiovascular Surgery, Heart and Diabetes Center NRW, Ruhr University, Bochum, Germany; ^2^Department of Social Sciences, University of Göttingen, Göttingen, Germany

**Keywords:** hypnosis, hypnotherapy, psychotherapy, heart, cardiac, surgery, intervention, adjunctive

## Abstract

Heart surgery patients are at high risk for psychological trauma and comorbid psychological disorders. Depression, anxiety, and post-traumatic stress disorders in this patient group are predictors of outcomes after cardiac surgery. Medical hypnosis is effective for non-pharmacologic prevention and treatment of psychological disorders and has been associated with improved health-related quality of life and better cardiovascular outcomes. This contribution makes note of evidence of the effectiveness of medical hypnosis in a discussion of the clinical experience with specific hypnotherapeutic tools and interventions from the perspective of the mental health team in one large cardiac center in Germany. Based on our experience, we encourage heart centers to educate their heart surgery care teams about the core concepts of medical hypnosis and to make hypnotherapeutic techniques available as an adjunctive therapy.

## 1 Introduction: medical hypnosis in heart surgery

This report explains why and how medical hypnosis has been used for treating perioperative burdens in heart surgery patients in one large heart center in Germany. It begins with a short review of psychiatric care needs in the perioperative setting for heart surgery patients, including current relevant guidelines for practitioners, then continues with an introduction to medical hypnosis and the evidence of its beneficial effects. It briefly introduces specific hypnotherapeutic techniques and their use in the heart surgery context. We close with our assessment of the contribution of medical hypnosis to mental health care for heart surgery patients and our recommendations.

Patients preparing for cardiovascular surgical interventions anticipate and sometimes experience their surgery as an extraordinary, life-threatening event over which they have no control. “No other organ evokes such strong emotions as the heart,” and this can lead to exceptional emotional and psychological burdens on heart surgery patients (Meffert, 2000, p. 280). Moreover, depression, anxiety, and post-traumatic stress disorders—all predictors of outcomes after cardiac surgery—are common in heart disease patients (Feuchtinger et al., 2014; Albus et al., 2019; Piepoli et al., 2020). Given these risks, psychological assessment and the provision of in-hospital psychological interventions as needed are recommended by international professional guidelines for various heart surgery patient groups (Dew et al., 2018; Chih et al., 2020; Kirklin et al., 2020). The German Cardiology Society calls also for including mental health professionals in heart failure units (Ertl et al., 2016).

Medical hypnosis started being used more commonly in hospital psychiatry in the 1970s. It encompasses many techniques that can be adapted to individual needs and hospital situations as an adjunctive treatment of somatic, psychological, and psychosomatic disorders (Häuser et al., 2016; Ginandes, 2017). Virtually all techniques use suggestions to positively influence emotions, cognitions, body sensations (pain, compression, etc.), and behaviors with or without inducing a trance state for strengthening their effects. Goals of medical hypnosis include relaxation, improving sleep, establishing feelings of security, heightening self-care capacity, pain management, accepting positive expectations about recovery, supporting post-operative mobilization, life-goal reorientation, adjusting body image after transplantation or mechanical circulatory support (MCS) implantation, and protecting against inadvertent negative suggestions. Many interventions can be standardized in the form of “scripts”; some examples are provided in the clinical discussion below.

Therapeutic suggestions are instructions from the therapist to the patient made in such a way as to circumvent an individual’s normal tendency to resist outside influences. Also common are indirect suggestions in the form of stories and metaphors to help patients unconsciously question and change dysfunctional expectations, ideas, and attitudes. Autosuggestions are suggestions that patients, after instruction, can repeat to themselves, aiding in relaxation and coping with long-term high-stress situations, anxiety, and pain.

The effect of suggestions can be strengthened if transmitted when the patient is in a trance state under which associative thinking, unwilled images, and memories are allowed to displace rational thinking. In this state, patients can still activate or relax their bodies as they wish, often in ways they could not consciously do, a phenomenon that can be exploited to help the patient via ideomotor signaling (see below). Importantly, patients in extreme medical situations often fall spontaneously into a “natural trance” (Hansen and Zech, 2019), making them susceptible to inadvertent negative suggestions from the hospital environment. Interventions can help patients guard against this.

In Table 1, we identify six random-controlled trials that yielded evidence of the effectiveness of medical hypnosis interventions specifically for adult cardiac surgery patients (Hart, 1980; Greenleaf et al., 1992; Ashton et al., 1997; Akgul et al., 2016; Scaglione et al., 2019; Garcia et al., 2020). These studies varied in sample size from 32 to 140 participants. Interventions were all conducted in-hospital. Beneficial associations for intervention groups were documented with anxiety, depression, procedural duration, perceived pain, medication consumption, duration of ventilator assistance, and recovery time. In addition, Weinstein and Au (1991) report in their controlled study of 32 patients undergoing angioplasty that the surgeon was able to keep the balloon inflated for the intervention group, which had received hypnosis, for a longer time and that the intervention group required less analgesic medication during the procedure; but they reported descriptive statistics only with no measure of statistical significance. Note that the studies in Table 1 also find no effect for some investigated outcomes. Two additional studies report finding no differences between intervention and control groups at all (Blankfield et al., 1995; Rousseaux et al., 2022). Several meta-analyses find statistically significant beneficial effects of a broader range of hypnosis interventions in patient groups undergoing also other kinds of surgery (Montgomery et al., 2002; Schnur et al., 2008; Ziehm et al., 2017). Barbero et al. (2018) describe five case studies.

**Table 1:** Randomized control trials (RCTs) of medical hypnosis interventions for the peri-operative care of cardiac surgery patients.

Study	Sample size	Surgical procedure	Intervention	Control(s)	Statistically significant (*p* < 0.05 or greater) benefit [Effect size, if reported]
Akgul et al. (2016)	*N* = 44	Coronary artery bypass grafting	Pre-operative, in-hospital single-session hypnosis by anesthesiologist	Pre-operative, in-hospital information on the surgical intervention by the same anesthesiologist	Lower anxiety [0.72, ANCOVA] Lower depression [0.65, ANCOVA] Fewer analgesic drugs doses Shorter ventilator assistance duration
Ashton et al. (1997)	*N* = 32	Coronary artery bypass surgery	Pre-operative self-hypnosis relaxation techniques	No therapy	Lower anxiety [corrected Hedges *g* = 0.80; 99% CI −0.17, 1.78; reported in Kekecs et al. (2014)] Pain medication required [corrected Hedges *g* = −0.86; 99% CI −1.84, 0.12; reported in Kekecs et al. (2014)]
Garcia et al. (2020)	*N* = 113	Atrial fibrillation	Hypnosis	Non-hypnotic relaxation suggestions and white noise through headphones	Lower mean pain score (VAS) Lower use of sedation during procedure Lower morphine dosage
Greenleaf et al. (1992)	*N* = 32	Coronary artery bypass surgery	Hypnosis	Routine care	Recovery time from surgery [Std. Mean Difference = 0.78; reported in Montgomery et al., 2002]
Hart (1980)	*N* = 40	Cardiopulmonary bypass surgery	Hypnosis	n/a	Lower anxiety [corrected Hedges *g* = 0.77; 99% CI −0.07, 1.62; reported in Kekecs et al. (2014)]
Scaglione et al. (2019)	*N* = 140	Undergoing atrial fibrillation ablation	In-hospital hypnotic communication for periprocedural analgesia	Conventional analgesia	Lower procedural-related anxiety Shorter perceived procedural duration

Weaknesses in the current state of the art include lack of participant blinding and the use of treatment as usual as control, both of which might introduce bias favoring interventions, and additionally a lack of data reporting that would enable calculation of treatment effects. An additional weakness is the lack of a clear empirical basis to differentiate the underlying causal effects of some general techniques such as imagination or seeding (see below). Are they best understood as a form of medical hypnosis specifically, as a trigger of unconscious placebo effects generally, or as a means to encourage beneficial rational-cognitive means of self-control?

## 2 Clinical experience

Our clinical experience using medical hypnosis for psychotherapeutic care arises from our work in one of Germany’s largest cardiac centers. The center performs about 4,000 major heart surgeries annually. Medical hypnosis has been available to patients since 2009. On staff are six psychologists who have completed a 2-year training program in hypnotherapy. Team sessions occur twice daily in addition to weekly sessions for intervention planning. A trained hypnotherapy instructor provides supervision sessions every 6 weeks.

About 75% of heart transplantation and MCS device patients and about 20% of other surgery patients receive medical hypnosis at some point during treatment at our center. Hypnotherapy is used to mitigate anxiety, pain, agitation, sleeplessness, specifically directed distress, and high general stress load. Sessions last between 30 and 60 min. The number of sessions per patient varies from 3 to 30, depending mostly on the duration of their hospital stay.

Goals of treatment are a heightened sense of well-being, lowered anxiety, lowered blood pressure, pulse stabilization, reduced muscle tension, and lowered use of analgesics, anxiolytics, benzodiazepines, and tranquilizers. Based on informal patient feedback we believe, not based on evidence, that the general level of satisfaction with hypnotherapy interventions is high. We believe that dissatisfaction is highest when patients are asked to conduct self-hypnosis; patients generally prefer to be guided by a therapist, but transferring self-hypnosis skills is a central therapy goal. In our clinical experience, dissatisfaction occurs also when therapy sessions are disturbed by outside noises or interruptions.

We do not conduct hypnotherapy if patients are taking psychoactive medications, during acute psychosis or postoperative delirium, and whenever communicating goals and instructions to the patient is not possible (e.g., language barriers). We instruct our therapists to be aware of the risk of unintentionally uncovering a repressed traumatic experience during trance, a very rare event in this patient population in our center.

## 3 Psychosocial care needs of heart surgery patients and interventions

Heart surgery patients’ predominant stressors and their common presentations of distress during the surgery process vary across three phases of hospital care: initial assessment and admission, shortly before and during the operation, and immediately after surgery (Tigges-Limmer et al., 2019; Salzmann et al., 2020). Typical sources of mental distress and common support tasks for medical hypnosis are summarized for each phase in Table 2, and these tasks dictate which hypnotherapeutic interventions are most common in each stage. The general hypnotherapeutic techniques we use are summarized below (*cf.* also Erickson et al., 1976; Lynn et al., 2010).

**Table 2:** Predominant mental health challenges for heart surgery patients and support tasks for mental health professionals using medical hypnosis.

Phase	Predominant mental health challenges for patients	Support tasks relevant for medical hypnosis
Initial assessment	Fear Pessimism regarding surgery outcome Attribution of meaning (subjective illness theory) Dysfunctional illness beliefs Maintenance of health-related personal control Adjusting to new social and work roles, incl. resetting life goals For PTSD patients: building a therapeutic relationship and a feeling of safety	Psychological assessment Crisis intervention in the event of psychological decompensation Finding meaning in the illness Encouraging optimism Coping with psychosomatic symptoms due to trauma, ambivalence, fear and worry, grief, anger, aggression, depressiveness, and sleep disorders Support of adherence, acceptance, and adjustment, e.g., facilitation of formation of new goals and roles Protection against inadvertent negative suggestions
Immediately before and during surgery	Acute stress reaction Fear Persisting anxiety post-surgery Feelings of helplessness Pain and physical discomfort After emergency procedures: apprising and adjusting to new situation	Crisis intervention in the event of psychological decompensation Coping with and reducing psychosomatic symptoms Relaxation techniques Protection against inadvertent negative suggestions
Immediately after surgery	Acute postoperative organic psychosyndrome Traumatic experiences Fear of pain of invasive procedures Fear of organ rejection or device failure Integration of new heart or device into body image Complications with recovery Physical discomfort	Support in processing an organic psychosyndrome, integrating the new heart or MCS device Coping with pain Psychological facilitation of recovery Coping with and reducing psychosomatic symptoms Relaxation techniques

Sources: Salzmann et al. (2020); Tigges-Limmer et al. (2019).

*Seeding* is the sending out of positive messages to the patient’s unconscious, often without a trance, with the possibility that these messages will “take root” and influence the patients’ own thoughts. This can be done, for example, during the therapist’s first interaction with the patient: “Hello, my name is […] and I am here for your well-being. I hope very much to help you feel safe and well in my presence…” Even this simple intervention is useful for establishing a trusting therapeutic relationship.

*Pacing and leading* are general techniques by which the therapist establishes a trusting rapport with the patient and methodically “talks” them to a mental place for therapeutic interaction. For patients experiencing acute anxiety, for example, the therapist focuses the patient’s attention by verbalizing the causes of anxiety and then acknowledging and affirming subjective feelings of fear and the need for help. Pacing is used also to slow breathing and racing thoughts, steering patients into feelings of greater security, well-being, and peace. An example of pacing might be: “I can see how nervous you are, and the situation really is nerve wracking. When we are nervous, we all breathe more shallowly than usual.” For leading: “Just take a deep breath in, and then breathe out deeply. As you breathe out, all the stress can be breathed out too. Calmly inhale fresh air and slowly exhale the used air out.”

*Imagination* is a technique to help patients develop their ability to use all their senses to vividly imagine mental pictures, emotions, future states, or other behaviors, for example with the “safe space” intervention by which the patient is invited to imagine a very specific space where they feel safe, calm, and relaxed. This space can be a favorite vacation spot, a beautiful place in nature, or a fantasized place of security. This technique can be used repeatedly as a relaxation technique by patients on their own; or, after inducing a trance, for the psychotherapeutic treatment of negative emotions or for crisis intervention. Future projection is a form of imagination by which patients imagine future experiences, thoughts, and feelings for the purpose of anchoring a strategy for full recovery. Patients are invited to spend time in an imagined, resource-rich state to enjoy the feeling of good health and to contemplate the possibilities of their improved life situation. Later, therapists can invite patients to remember the future projection they did before the operation to reactivate their healing strategies. Some patients have dysfunctional cognitions, such as helplessness, that impair their sense of self-worth and security and can increase anxiety and impede healing. One alleviating technique is the “inner healer” form of imagination: in this script, the patient is invited to imagine a walk in some peaceful place where they approach from a distance a wise and friendly doctor, who is installed as the patient’s inner healer with the power to give information about what the patient needs. This also helps patients who are confronting feelings of passivity and loss of control.

*Anchoring* works by fixing a central message or suggestion to an object or event, serving as a memory aid. The scripted “hand anchor” intervention helps patients facing surgery anxiety (Tigges-Limmer et al., 2018). Pre-surgery, the patient is led in trance to examine with their inner eye each finger and thumb of the left hand, associating specific resources with each: optimism and hope, trust and protection against negative suggestions, future-orientation, social supports, and the safe place. The hand serves here as a memory aid, and the cultural significance of each finger can be utilized, e.g., “thumbs up” is associated with positive feelings or hope.

Through *ideomotor signaling*, a person in a trance state communicates by translating thoughts into muscle movement without “willing” the muscle to move. Patients can learn to give ideomotor signals to their nurses and doctors. A specific use is the “emergency finger signal”: one finger is ideomotorically trained to move whenever the patient has the thought, “I need emergency help.” This can help alleviate the anticipated fear of patients who had experienced not being able to communicate during a traumatic surgery event. Another example is ideomotor arm “levitation,” which we use to estimate a patient’s hypnotizability.

*Utilization* is the use of memories, objects, stimuli, symptoms, character traits, etc. for activating therapeutically useful resources. Utilization is combined with *focusing* such that the therapist invites the patient to concentrate their attention on something to utilize its meaning. For example, patients who are distressed by the noise made by breathing machines, intensive care unit monitors, or by their MCS can be invited to focus on these sounds to reframe (see below) their meaning: “the language machines use to talk to medical personnel when they need to help me, so when I hear the noise, I can relax.” In this way, annoying sounds can be utilized in a relaxation technique.

*Reframing* refers to strategies for expanding the perspectives patients use to impart meaning to their experiences. The therapist works with the patient to look at the perspective or “frame” through which the patient attributes meaning. Negative framing can generate problematic emotions and behaviors, and through reframing, the meanings that the patient imparts to experiences can be turned positive. The “journey to the heart” script helps patients reframe their worry to a sense of caregiving. In trance, patients are invited to make gentle contact with their heart in a quiet space, listening with growing self-confidence to what it might wish to say and perhaps taking the opportunity to respond (Tigges-Limmer and Schmid-Ott, 2012).

*Dissociation* is a mechanism for protecting against overwhelming strain in potentially traumatic situations. It works by blocking out areas of conscious thought. It can be used to suppress pain and is useful for preparing patients for potentially traumatizing situations. With this technique, patients are invited to “remove” themselves to a place where they feel secure and can control the sources of their fear, for instance by imagining watching themselves in the anxiety-producing situation on a “video screen” in their mind with the power to rewind, pause, or fast forward to regain a sense of control. *Association* works by connecting all areas of conscious thought to achieve more complete understanding and to use conscious thought in a positive way. For example, patients in a trance state are invited to approach a source of anxiety with an open mind for the purpose of finding a means of emotional accommodation. This is used post-surgery, e.g., with transplant patients in the “greeting the new heart” script (Tigges-Limmer and Gummert, 2010).

*Glove anesthesia* is a technique that gives patients the feeling of being able to locally anesthetize specific body areas. The patient is given the suggestion of wearing a glove soaked in a powerful local anesthesia that soaks into the skin and nerves of the hand, numbing it. Patients are instructed that this numbness can be transferred to other parts of the body by laying their “gloved” hand on it. This is used, e.g., during post-op removal of thorax drainage tubes, a procedure that is often anticipated as painful.

Finally, we also make extensive use of suggestive *metaphors*. Interventions include those that help patients impart meaning to medical procedures and necessities. These should be individually tailored. For example, an automobile enthusiast might enjoy thinking of his new pacemaker as more “turbo horsepower,” and gardeners might be helped by the image of “pulling weeds” for wound cleansing.

## 4 Individual factors that influence the effectiveness of medical hypnosis

Patient characteristics can moderate the effectiveness of medical hypnosis. Some work has looked at hypnotizability and its associations with factors like “cognitive phenotypes” and interoceptive awareness that may be in part genetically preconditioned (Diolaiuti et al., 2019; Faerman and Spiegel, 2021). Hypnotizability is a measurable trait that influences the effectiveness of medical hypnosis techniques that make use of trances, perhaps especially for pain control (Santarcangelo and Consoli, 2018), but it is important to note that many techniques of medical hypnosis can be used without inducing a trance. In our clinical experience, we have noticed that the expectation of positive benefit from hypnosis seems to be associated with treatment success, suggesting that hypnosis may in some cases be augmented by placebo effects or work by triggering placebo effects. Apparently, the higher the expectation, the higher the effectiveness; this expectation seems more likely to be linked to past experiences of success than to genetic dispositions.

Also, individual-level differences in the presentations of psychological distress and on individual paths to successful treatment are relevant for the success of medical hypnosis. Indeed, an individual-level focus is by necessity central to medical hypnosis, because treatment effectiveness depends on the therapist being able to use suggestions, anchors, and metaphors attuned to an individual’s unique set of feelings and experiences. There is variability also in the sources of psychological stress for heart surgery patients and in how patients present their felt psychological distress. Given these conditions of treatment success, the tools of medical hypnosis must be diverse and implementable in various combinations to fit with every patient’s individual support needs.

## 5 Discussion

Medical hypnosis has been used in our clinic for heart surgery patients because it can be used in all phases of surgical care, is well suited for addressing overwhelming emotions, can also can be conducted when two-way verbal communication and cognitive function are impaired, can be quite rapid in its effects, can be learned by patients for some forms of self-help, and includes many techniques that are easily combined into personalized therapy toolkits. It buffers the difficult, scary, and challenging experience of heart surgery for our patients in a way that can be easily adjusted to their support needs and coping styles. Because its focus is on activating patient resources, we have seen few negative side effects.

In our experience, medical hypnosis brings benefits for everyone involved in heart surgery. Mental health professionals gain new tools to use in situations where other techniques are hampered. Surgeons and nurses benefit because their patients are better able to self-regulate their anxiety, stress, and pain. Family caretakers benefit in this way, too, but they also can receive medical hypnosis from clinic staff for their own personal advantage. Patients benefit in the ways noted in the review of the evidence above. Finally, as medical hypnosis has been shown to be associated with lower procedure duration, lower recovery time and lower medication use, it is associated also with cost-related benefits.

Given these advantages, we encourage heart surgery care teams to consider educating all members about the basic concepts of medical hypnosis. Especially important in our view is sensitizing personnel about hypnotizability and the potential impact of negative suggestions. All team members can learn low-complexity, non-trance interventions such as seeding and metaphors. We also encourage mental health professionals working in these teams to learn methods of trance-induction and to use a wide range of medical hypnosis techniques.

## Data availability statement

The original contributions presented in the study are included in the article/supplementary material, further inquiries can be directed to the corresponding author. English translations of German scripts are available from the corresponding author.

## Author contributions

KT-L: Writing – original draft, Writing – review & editing. YB: Conceptualization, Writing – original draft, Writing – review & editing. YW: Conceptualization, Writing – original draft, Writing – review & editing. SSG: Writing – original draft, Writing – review & editing. JG: Project administration, Resources, Supervision, Writing – review & editing.
